# Xanthones from the Roots of *Moutabea guianensis* Aubl.

**DOI:** 10.3390/molecules20010127

**Published:** 2014-12-23

**Authors:** Haroldo da S. Ripardo Filho, Luidi C. Pacheco, Edinaldo da S. Andrade, Marivaldo José C. Correa, Railda Neyva M. Araújo, Giselle Maria S. P. Guilhon, Joyce Kelly R. da Silva, Lourivaldo S. Santos

**Affiliations:** 1 Instituto de Ciências Exatas e Naturais, Universidade Federal do Pará-UFPA, Rua Augusto Corrêa, S/N, 66075-900 Belém-PA, Brazil; E-Mails: ripardofilho@ufpa.br (H.S.R.F.); luidicp@hotmail.com (L.C.P.); edinaldometalurgico@hotmail.com (E.S.A.); majocost@ufpa.br (M.J.C.C.); raildamoreira@hotmail.com (R.N.M.A.); giselle@ufpa.br (G.M.S.P.G.); 2 Programa de Pós-Graduação em Biotecnologia, Instituto de Ciências Biológicas, Universidade Federal do Pará-UFPA, Rua Augusto Corrêa, S/N, 66075-900 Belém-PA, Brazil; E-Mail: joycekellys@ufpa.br

**Keywords:** *Moutabea guianensis*, Polygalaceae, xanthones, antioxidant activity

## Abstract

The phytochemical investigation of *Moutabea guianensis* roots led to the isolation of five polyoxygenated xanthones, including two new ones named moutabeone B (1,8-dihydroxy-4,5,6,7-tetramethoxyxanthone) and moutabeone C (1-hydroxy-4,5,6,7,8-pentamethoxyxanthone), along with the three known xanthones, 1,8-dihydroxy-4,6-dimethoxyxanthone, 1,8-dihydroxy-4,5,6-trimethoxyxanthone and augustin A (1,8-dihydroxy-4,6,7-trimethoxyxanthone). Structural characterization of all compounds was established on the basis of spectroscopic methods, mainly 1D and 2D nuclear magnetic resonance (NMR) and comparison with literature data. The antioxidant activity of compounds was tested through a thin layer chromatography (TLC) bioautography assay using 1,1-diphenyl-2-picryl-hydrazyl radical (DPPH**·**) as detection reagent. All tested compounds were more active (DL < 0.13–0.03 µg) than Trolox (DL < 0.15 µg), used as reference standard.

## 1. Introduction

Xanthones are secondary metabolites commonly occurring in a few higher plant families and their pharmacological activities have aroused great interest [1]. An important natural source of these compounds is the Polygalaceae family which includes about 22 genera and 1,300 species [2]. The genera *Monnina*, *Bredemeyera*, *Securidaca* and *Polygala* are the main sources of xanthones in the Polygalaceae [3,4,5]. On the other hand, there is only one report of the isolation of xanthones from the genus *Moutabea*, which described earlier chemical investigation of *M. guianensis* [6]. In continuation of the study of *M. guianensis*, the isolation and structural elucidation of two new xanthones and also other three known xanthones is now reported.

## 2. Results and Discussion

**Figure 1:**
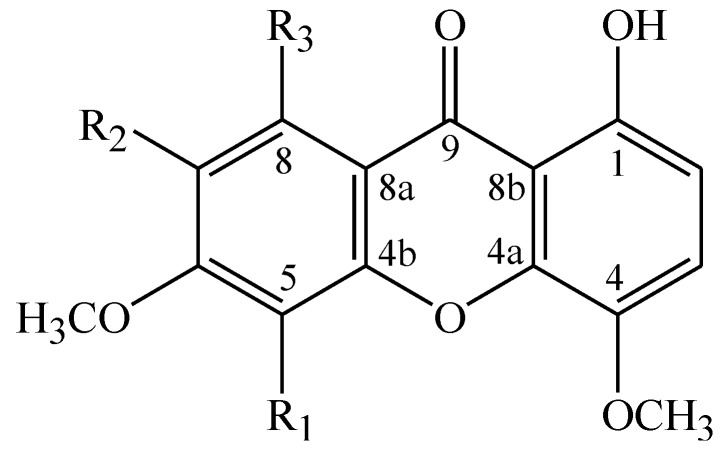
Xanthones from *Moutabea guianensis*.

This chemical investigation led to the identification of five compounds (Figure 1); among these, three had already been isolated from other species and two are reported here as new compounds. The known compounds were identified as 1,8-dihydroxy-4,6-dimethoxyxanthone (**1**) [7], 1,8-dihydroxy-4,5,6-trimetoxyxanthone (**3**) [8], and 1,8-dihydroxy-4,6,7-trimethoxyxanthone (**4**) [9], by comparison of their spectral data with those in the literature. The new compounds were identified as 1,8-dihydroxy-4,5,6,7-tetramethoxyxanthone (**2**) and 1-hydroxy-4,5,6,7,8-pentamethoxyxanthone (**5**).

**Table:** 

	R_1_	R_2_	R_3_
1	H	H	OH
2	OCH_3_	OCH_3_	OH
3	OCH_3_	H	OH
4	H	OCH_3_	OH
5	OCH_3_	OCH_3_	OCH_3_

**Figure 2:**
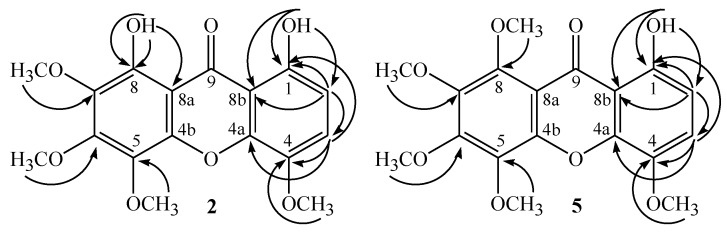
HMBC correlations (H→C) of compounds **2** and **5**.

Compound **2** was obtained as a yellow solid and its molecular formula (C_17_H_16_O_8_) was determined based on the HRESIMS data (*m/z* 349.0921 [M+H]^+^ calcd. for C_17_H_17_O_8_^+^, 349.0923). The ^1^H-NMR spectrum (Table 1) exhibited two doublets in the aromatic region, one at δ_H_ 6.73 (1H, *J* = 9.0 Hz) and the other at 7.26 (1H, d, *J* = 9.0 Hz) assigned to one pair of *ortho*-coupled hydrogens (H-2 and H-3), four signals of four methoxyl groups at δ_H_ 3.94–4.16 (3H each, *s*) and two singlets (δ_H_ 11.29 and 11.77 assigned to phenolic OH groups at C-1 and C-8 of a xanthone, which are strongly dishielded due to the hydrogen bonds to the carbonyl group (C-9). The ^13^C-NMR and DEPT spectra (Table 1) showed 17 signals corresponding to a 3,8-dihydroxytetramethoxyxanthone. The carbon signal at δ_C_ 185.5 is characteristic for a doubly chelated carbonyl (1,8-di-OH) [10] and three of four methoxyl signals (57.6, 61.2, 61.7 and 61.9) were typical of di-*ortho*-substituted methoxyl groups (δ_H_ > 60) [11]. The ^13^C-NMR further shows two methine carbons (δ_C_ 109.3 and 121.3), ten non-hydrogenated carbons, two of which have no oxygen substituent (δ_C_ 103.9 and 107.8) and eight of which have an oxygen substituent (δ_C_ 132.8–154.9). The assignments of the signals at δ_C_ 154.1 (C-1, ^2^*J*_C,H_), 109.3 (C-2, ^3^*J*_C,H_) and 107.8 (C-8b, ^3^*J*_C,H_) were confirmed based on the long-range correlations (HMBC) with signal at δ_H_ 11.29 (OH-1). Long-rang correlations of hydroxyl hydrogen at δ_H_ 11.8 (OH-8) with the signals at δ_C_ 135.7 (^3^*J*_C,H_), 150.2 (^2^*J*_C,H_) and 103.9 (^3^*J*_C,H_) permitted the assignment of these signals to C-7, C-8 and C-8a, respectively. Furthermore, it can be observed correlation of the methoxyl hydrogen at δ_H_ 3.94 with the carbon signal at δ_C_ 135.7 (C-7, ^3^*J*_C,H_), indicative that this methoxyl group is at position 7. Assigments of the signals at δ_C_ 132.8 (C-5) and δ_C_ 154.9 (C-6) was supported by the ^3^*J*_C,H_ correlations with δ_H_ 4.00 (OCH_3_-5) and 4.16 (OCH_3_-6), respectively and confirmed by NOE effect observed in the NOE-diff experiment, which revealed spatial interactions between δ_H_ 3.94 (OCH_3_-7) and δ_H_ 4.16 (OCH_3_-6), and also between δ_H_ 4.16 (OCH_3_-6) and δ_H_ 4.00 (OCH_3_-5). The other chemical shifts were assigned by HETCOR (^1^*J*_C,H_) and HMBC (^2,3,4^*J*_C,H_) spectra. HMBC correlations are shown in Figure 2. The structure of **2** was then identified as 1,8-dihydroxy-4,5,6,7-tetramethoxyxanthone and named moutabeone B. This compound is a new natural product.

**Table 1:** ^1^H-NMR (300 MHz) and ^13^C-NMR (75 MHz) spectral data for compounds **2** and **5** in CDCl_3_.

Positions	Compound 2		Compound 5	
	δ_H_ (*J* in Hz)	δ_C_ and DEPT	δ_H_ (*J* in Hz)	δ_C_ and DEPT
**1**		154.1, C		154.9, C
**2**	6.73, d, 9.0	109.3, CH	6.70, d, 8.8	109.0, CH
**3**	7.26, d, 9.0	121.3, CH	7.22, d, 8.8	120.4, CH
**4**		140.3		139.8, C
**4a**		145.6, C *		145.1, C
**4b**		145.8, C *		147.7, C
**5**		132.8, C		137.4, C
**6**		154.9, C		153.1, C
**7**		135.7, C		149.2, C
**8**		150.2, C		143.2, C
**8a**		103.9, C		111.2, C
**8b**		107.8, C		109.4, C
**9**		185.5, C		181.6, C
**4-OCH_3_**	3.97, s	57.6, CH_3_	3.96, s	57.6 , CH_3_
**5-OCH_3_**	4.00, s	61.7, CH_3_	4.10, s	61.9, CH_3_
**6-OCH_3_**	4.16, s	61.9, CH_3_	4.14, s	61.7, CH_3_
**7-OCH_3_**	3.94, s	61.2, CH_3_	3.99, s	62.1, CH_3_
**8-OCH_3_**			3.94, s	62.8, CH_3_
**1-OH**	11.29, s		12.39, s	
**8-OH**	11.77, s			

* Signals can be interchanged.

Compound **5** was also obtained as a yellow solid. Its molecular formula was deduced to be C_18_H_18_O_8_ from its [M+H]^+^ peak at *m/z* 363.1077 (calcd. for C_18_H_19_O_8_^+^, 363.1079) in the HRESIMS. The ^1^H- and ^13^C-NMR data of compound **5** were very similar to those reported for compound **2**. The main difference is the presence of five methoxyl groups instead of four and the presence of only one hydroxyl group instead of two, indicating that compound **5** could have a methoxyl group instead a hydroxyl group at C-8. Moreover, the B ring of **5** is similar to the totally substituted ring of 1-hydroxy-3,5,6,7,8-pentamethoxyxanthone [10], so the ^13^C-NMR data of C-4b, C-5, C-6, C-7, C-8 and C-8a were very close. Compound **5** was thus identified as 1-hydroxy-4,5,6,7,8-pentamethoxyxanthone, a new natural product, named moutabeone C. HMBC correlations observed for compound **5** are shown in Figure 2 and confirmed the structure.

**Figure 3:**
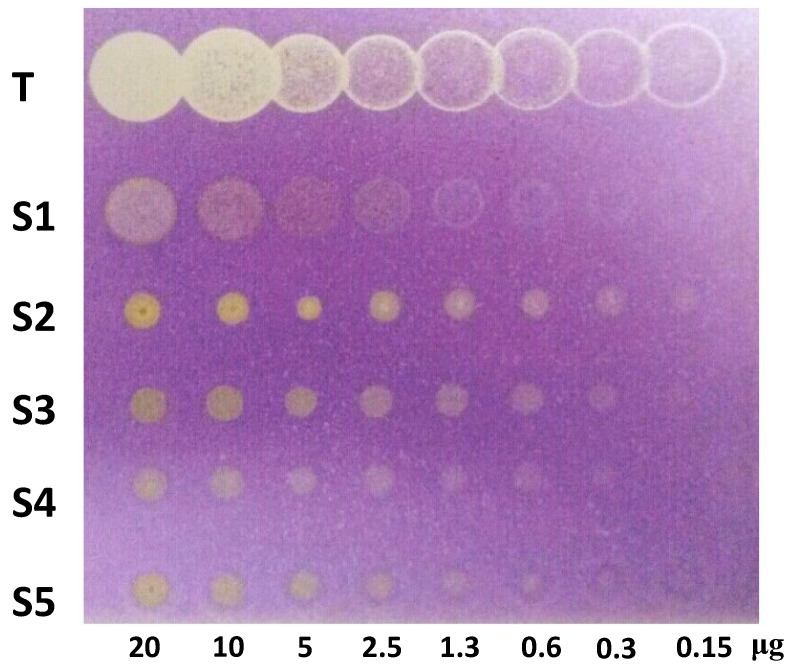
TLC plate showing the antioxidant activity by compounds (**1**–**5**) and Trolox (T). Substances along the y-axis and concentration along the x-axis.

Thin-layer chromatography, combined with both biological and chemical detection methods, is an effective and inexpensive technique for the study of bioactive compounds [12]. In the antioxidant assay, the samples showed different values for the detection limit (DL), which were compared with a reference antioxidant Trolox (DL < 0.15 µg) (Figure 3). The increasing order of activity based on the observed detection limits was **1** (1.3 µg) < **5** (0.6 µg) < **3** and **4** (0.3 µg) < **2** (<0.15 µg). The detection limit of **2** was equivalent to Trolox (<1.5 µg).

Xanthones have been reported to inhibit lipid peroxidation, antioxidant activity, neuroprotective properties [13]. The xanthone nucleus is known as 9-xanthenone or dibenzo-γ-pyrone and its antioxidant activity depends of specific substituents and their positions to the basic structure of each molecule [14]. The xanthone **1** has only two substituents in the B ring and showed less activity. However, the highest activity was observed for xanthone **2**, which has four substituents in the B ring (one hydroxyl and three methoxyl groups). The increase in antioxidant activity may be attributed to the number of hydroxyl and methoxyl groups, which are strong protons and electrons donors, respectively [15,16].

## 3. Experimental Section

### 3.1. General

NMR spectra were recorded on a Mercury-300 NMR spectrometer (Varian, Palo Alto, CA, USA; 300 MHz and 75 MHz for ^1^H- and ^13^C-, respectively) using TMS as internal standard. HRESIMS was carried out on a Waters Xevo G2-S QTof/Tof spectrometer (Milford, MA, USA). IR was carried out on a Shimadzu Prestige 21 (Tokyo, Japan). Column chromatography was performed on silica gel 60 (70–230 mesh, Macherey-Nagel, Düren, Germany). Precoated sheets of silica gel with UV254 indicator (thickness 200 µm) were used for TLC (Sorbent technologies, Norcross, GA, USA). Spots were visualized either with a UV lamp (254 nm) or by spraying with aqueous H_2_SO_4_ (15%) saturated with CeSO_4_ solution, followed by heating. Semipreparative HPLC was carried using a Varian liquid chromatograph with UV detector model ProStar 335; using a Phenomenex Gemini (Torrance, CA, USA) C18 column (250 mm × 10 mm, 5 μm).

### 3.2. Plant Material

The roots of *M. guianensis* were collected in the experimental field of Embrapa Amazônia Oriental, located in Belém, Pará State, on March 2012. A voucher specimen (195862) was kept in the Herbarium MG of the Museu Paraense Emílio Goeldi (MPEG). Roots were dried under forced air circulation at 40 °C for five days and powdered in a knife mill.

### 3.3. Extraction and Isolation

Dried and powered roots of *M. guianensis* (928 g) were submitted to successive extractions with hexane (3 L), ethyl acetate (3 L) and methanol (3 L) at room temperature for five days each solvent. After removal of the solvent *in vacuo*, the hexane, ethyl acetate and methanol extracts were obtained, respectively. The ethyl acetate extract (2.5 g) was subjected to column chromatography (CC) on silica gel, using hexane and increasing proportions of EtOAc as eluents, collecting 25 fractions of 125 mL each. The fractions 1–5 eluted with 95:5 (hexane/EtOAc) were combined according to TLC to afford fraction A (185 mg). Fraction A was purified by semi-preparative HPLC (ACN/H_2_O, 1:1, isocratic system and flow rate 4.7 mL/min) yielding compounds **1** (8 mg), **2** (25 mg), **3** (7 mg), **4** (13 mg) and **5** (7 mg) which showed chromatographic peaks with retention times 21.57, 22.8, 28.59, 31.93 and 37.01 min, respectively.

### 3.4. Characterization

^13^C-NMR (75 MHz, CDCl_3_) chemical shifts of compounds **1**, **3** and **4** obtained in this work: 

*1,8-Dihydroxy-4,6-dimethoxyxanthone* (**1**), δ_C_ 162.8 (C1), 97.9 (C2), 167.4 (C3), 93.1 (C4), 157.7 (C4a), 154.1 (C4b), 139.9 (C5), 120.4 (C6), 109.3 (C7), 145.4 (C8), 108.1 (C8a), 184.6 (C9), 102.8 (C8b), 57.4 (4-OCH_3_), 55.9 (5-OCH_3_).

*1,8-Dihydroxy-4,5,6-trimethoxyxanthone* (**3**), δ_C_ 154.3 (C1), 109.4 (C2), 121.6 (C3), 140.3 (C4), 145.7 (C4a), 149.1 (C4b), 129.2 (C5), 160.5 (C6), 95.1 (C7), 158.1 (C8), 107.8 (C8a), 184.9 (C9), 107.2 (C8b), 57.3 or 56.5 (4-OCH_3_), 60.9 (5-OCH_3_), 57.3 or 56.5 (6-OCH_3_).

*1,8-Dihydroxy-4,6,7-trimethoxyxanthone* (augustin A) (**4**), δ_C_ 154.0 (C1), 109.2 (C2), 120.0 (C3), 139.9 (C4), 145.4 (C4a), 153.3 (C4b), 91.2 (C5), 160.7 (C6), 132.2 (C7), 153.6 (C8), 103.2 (C8a), 184.9 (C9), 107.9 (C8b), 57.3 (4-OCH_3_), 56.5 (6-OCH_3_), 60.9 (7-OCH_3_). 

### 3.5. Antioxidant Capacity by DPPH Assay

The antioxidant activity of xanthones was performed by TLC bioautography analysis [17]. The compounds were solubilized in chloroform at concentration of 2 mg/mL and diluted successively. For the assay, 10 µL of each solution were applied on the TLC plate corresponding to 20, 10, 5, 2.5, 1.3, 0.6, 0.3 and 0.15 µg/spot. The plate were sprayed with a DPPH solution 0.5 mM methanol solution for derivatization. Bands with the DPPH**·** scavenging activity were observed as white or yellow bands on a purple background. The antioxidant Trolox (Sigma Aldrich, St. Louis, MO, USA) was used as reference standard.

## 4. Conclusions

The chemical study of the roots of *M. guianensis* resulted in the isolation of five xanthones by chromatographic methods. The structures of the isolated compounds were proposed based on NMR methods. Compounds 1,8-dihydroxy-4,5,6,7-tetramethoxyxanthone (**2**), 1-hydroxy-4,5,6,7,8-pentamethoxyxanthone (**5**) were identified as new natural products. Compound 1,8-dihydroxy-4,6,7-trimethoxyxanthone (**4**) was isolated for the first time in the family Polygalaceae. This study shows that *Moutabea* genus is an important source of natural xanthones. All isolated compounds showed significant antioxidant activity in the thin layer chromatography (TLC) bioautography assay, equivalent to Trolox used as reference standard.
